# Eco-friendly formulations of essential oil-based silica nanocarriers as antibacterial agents

**DOI:** 10.3389/fbioe.2026.1903269

**Published:** 2026-08-28

**Authors:** Chiara Artusi, Ilaria Zanoni, Daniele Ghezzi, Gabriela Graziani, Marina Serantoni, Magda Blosi, Stefano Fedi, Martina Cappelletti, Anna Luisa Costa, Enrico Sassoni, Simona Ortelli

**Affiliations:** 1 CNR-ISSMC, National Research Council - Institute of Science, Technology and Sustainability for Ceramics, Faenza, Italy; 2 Department of Pharmacy and Biotechnology, University of Bologna, Bologna, Italy; 3 Department of Human Sciences and Quality of Life, San Raffaele Rome University, Rome, Italy; 4 DICAM, Department of Civil, Chemical, Environmental and Materials Engineering, University of Bologna, Bologna, Italy

**Keywords:** antibacterial activity, encapsulation, essential oil, Safe and Sustainable by Design, SiO_2_ nanocarrier

## Abstract

The industrial application of essential oils (EOs) is hindered by their high volatility, instability and poor solubility. Encapsulation represents an effective strategy to overcome these limitations by enhancing protection, enabling controlled release, and improving functional properties. In this study, we developed an aqueous-based sol–gel synthesis, combined with the interfacial oil miniemulsion method, to encapsulate blends of lavender (LO), thyme (TO), and cinnamon (CO) essential oils within silica nanocarriers, in order to hinder EOs volatility and instability, as well as control their release. It was also investigated whether combining LO with other essential oils (TO and CO) could provide complementary or synergistic effects. The process was conducted predominantly in water, using TEOS as silica precursor and a non-ionic surfactant under controlled conditions, aiming to reduce solvent consumption and process-related hazards in line with Safe and Sustainable by Design approach. Physicochemical characterization (SEM, FTIR, TGA, DLS, HPLC) confirmed the formation of spherical silica nanocarriers with diameters of 55–85 nm, narrow size distributions (PdI < 0.15), and encapsulation efficiencies of 93%–94%, corresponding to loading capacities of 52 wt. %. Release studies showed a sustained behavior, with release limited to 15%–26% over 48–168 h, depending on the formulation, and kinetic modelling indicated predominantly diffusion-controlled release. Antibacterial assays against *Escherichia coli* and *Staphylococcus aureus* showed that encapsulated EO blends preserved their antibacterial activity. SiO_2_ nanocarriers loaded with LO showed MIC values to 25% for *E. coli* and 3.125% for *S. aureus*. Notably, the oil blends improved LO performance also within the encapsulated system, with MIC values of 6.25% and 0.78% (for LO and CO), and 6.25% and 1.56% (for LO and TO) against *E. coli* and *S. aureus*, respectively. Overall, this study demonstrates the feasibility of integrating aqueous sol–gel processing with interfacial miniemulsion to fabricate silica nanocarriers for essential oil delivery, achieving enhanced stability, controlled release behaviour, and preserved antibacterial performance, with promising implications for sustainable formulations.

## 1 Introduction

Essential oils (EOs) are volatile, aromatic compounds produced as secondary metabolites by plants and have been used for centuries in traditional medicine, perfumery, and food preservation. Their complex chemical composition, including terpenes, terpenoids, phenylpropenes, alcohols, and aldehydes, confers a wide range of biological activities, including well-documented antimicrobial, antioxidant, and anti-inflammatory properties (Bravo Cadena et al., 2018). Many of these constituents exert antibacterial activity by interacting with microbial membranes, increasing permeability and disrupting cellular integrity. Moreover, synergistic blending of different EOs may broaden the antimicrobial spectrum and reduce the effective concentration of individual components. Among commonly studied EOs, lavender oil (LO) is widely recognized for its anti-inflammatory and antioxidative properties and moderate antimicrobial activity when used individually (But et al., 2023; Kajjari et al., 2022). However, combining LO with thyme oil (TO) or cinnamon oil (CO) significantly enhances antimicrobial performance due to synergistic interactions among key constituents such as thymol, cinnamaldehyde, and linalool (Nair et al., 2022; Nazzaro et al., 2013).

In recent decades, growing concerns about antimicrobial resistance and the demand for natural and sustainable alternatives to synthetic preservatives have renewed interest in EOs as multifunctional bioactive agents (Liao et al., 2021). Despite their potential, the direct application of EOs in industrial sectors such as biomedicine, construction and heritage conservation is hampered by several intrinsic limitations. High volatility results in rapid evaporation and loss of activity, while sensitivity to light, oxygen, and heat leads to chemical degradation of active components (He et al., 2025). In addition, their poor solubility in aqueous systems restricts their incorporation into water-based formulations, such as beverages, topical gels, or pharmaceutical suspensions. These limitations highlight the need for delivery systems capable of protecting EOs, preserving their properties, increasing their stability, and ensuring controlled release at the site of action (Fakhariha et al., 2025).

In this regard, various encapsulation technologies have been explored to address these challenges, including emulsification, spray drying, freeze drying, precipitation, and gelation techniques (Nair et al., 2022; Yammine et al., 2024; Weisany et al., 2024; Phanse and Chandra, 2024; Lammari et al., 2020). Depending on the selected strategy, different carriers, such as liposomes, cyclodextrins, polymeric matrices, chitosan nanoparticles and inorganic nanoparticles, can be employed, each offering specific structural features that influence protection efficiency, stability, and release behaviour (Kfoury et al., 2015; Natrajan et al., 2015; Paiva-Santos et al., 2022; Ruiz-Rico et al., 2017; Sebaaly et al., 2015; Reale et al., 2024; Wang et al., 2022). Among the different encapsulation platforms investigated for essential oils, silica-based nanocarriers have attracted considerable attention owing to their high chemical stability, tunable porosity and ability to protect volatile compounds while enabling controlled release (Ortelli et al., 2016; Hedayati et al., 2024).

Previous studies have successfully demonstrated the encapsulation of individual essential oils, including thyme, cinnamon, oregano and other antimicrobial oils, in silica nanocarriers prepared by sol–gel and interfacial miniemulsion approaches (Presentato et al., 2020; Jobdeedamrong et al., 2018). For instance, Ruiz-Rico et al. (2017) demonstrated that immobilizing essential oil components on silica surfaces could enhance their baseline antimicrobial activity against foodborne pathogens. Similarly, Presentato et al. (2020) developed mesoporous silica nanoparticles specifically tailored for the controlled release of biocides in stone preventive conservation, proving the spatial utility of inorganic frameworks in protecting active compounds from rapid volatilization. Moreover, Fakhariha et al. (2025) demonstrated that the nanoencapsulation of Sage and Thyme essential oils in silica hollow nanospheres and hollow polymer nanocapsules enhanced their stability, release behavior, and antimicrobial properties. Despite these advances, two critical limitations consistently restrict the industrial translation and ecological viability of current encapsulation technologies, defining a clear gap in the state of the art. First, conventional interfacial miniemulsion protocols rely heavily on the addition of hydrophobic organic co-solvents (such as hexadecane) to suppress Ostwald ripening, alongside cationic surfactants like cetyltrimethylammonium bromide (CTAB) to template the silica network (Jobdeedamrong et al., 2018; Zhong et al., 2021). From a green chemistry perspective, these auxiliary chemicals significantly increase the toxicological footprint and environmental hazard of the processing phase. Consequently, there is an urgent need to pioneer alternative silica synthesis routes that strictly comply with the Safe and Sustainable by Design (SSbD) framework—achieving solvent-free, eco-friendly formulations without sacrificing the structural integrity and high loading performance of the nanocarriers (Brunelli et al., 2025). Second, and perhaps most crucially, literature has thoroughly documented that binary or ternary combinations of essential oils in their free form can yield powerful synergistic antimicrobial effects due to complementary mechanisms, such as concurrent cell wall disruption and cytoplasmic leakage (Bassolé and Juliani, 2012). However, the transposition of these multi-component synergistic blends into rigid inorganic nanocarriers remains scarcely studied. When multiple volatile compounds with highly diverse polarities and chemical structures (e.g., the aromatic aldehyde cinnamaldehyde vs. the terpenoid alcohol linalool) are co-encapsulated, they face competitive surface interactions within the confined spaces of a developing silica shell. This chemical competition can profoundly alter their individual retention capacities and release kinetics, potentially compromising or nullifying the original synergistic effect. To date, a systematic investigation into whether a purely aqueous silica nanocarrier can efficiently co-encapsulate synergistic EO blends while actively preserving, or even amplifying, their combined antibacterial dynamics has not been widely reported.

To address these challenges, the primary objective of this work was to develop an integrated aqueous sol–gel/interfacial miniemulsion strategy inspired by SSbD principles, to produce SiO_2_ nanocarriers encapsulating blends of essential oils, thereby preserving their antibacterial activity by improving stability and enabling a more sustained release. Specifically, lavender oil was chosen as a benchmark and combined in binary systems with cinnamon or thyme oils to systematically evaluate, for the first time, how the silica matrix modulates the simultaneous release, kinetic diffusion, and combined antimicrobial efficacy against both Gram-negative (*Escherichia col*i) and Gram-positive (*Staphylococcus aureus*) model strains. This investigation successfully demonstrates an eco-friendly pathway to mitigate the high volatility of EOs while optimizing their long-term functional performance for advanced applications.

## 2 Materials and methods

### 2.1 Chemicals and reagents

Tetraethyl orthosilicate (TEOS, 98%), t-Octylphenoxypolyethoxyethanol (Triton X-100, laboratory grade), Polysorbate 80 (Tween 80, laboratory grade) were purchased from Sigma Aldrich (Milan, Italy). Lavender oil (LO) obtained from Lavandula Officinalis, cinnamon oil (CO) obtained from Cinnamonum Cassia, and thyme oil (TO) obtained from Thymus Vulgaris were purchased from Erboristeria Magentina S.r.l (Turin, Italy), with a purity of 100%. Ethanol (HPLC grade, ≥99.8%) was purchased from PanReac AppliChem ITW Reagents S.R.L. (Monza, Italy), Acetonitrile (ACN) (HPLC grade, ≥99.9%) was purchased from Carlo Erba Reagents (Milan, Italy). Ultra-pure water was produced by a MilliQ® water purifier system (Millipore, resistivity ≥18.2 MΩ⋅cm, total organic carbon ≤3 μg/L).

### 2.2 Encapsulation of essential oils in silica nanocarriers

**FIGURE 1:**
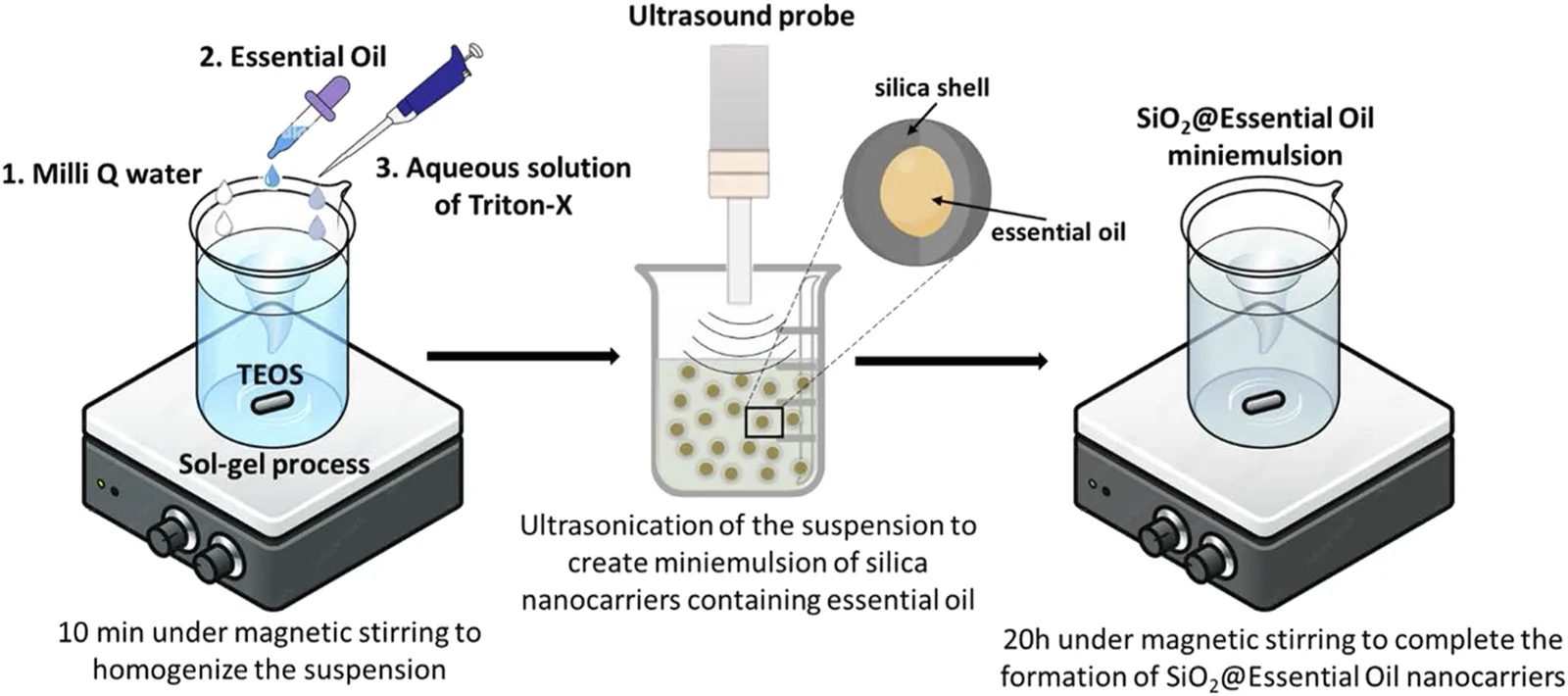
Schematic representation of the synthetic procedure of silica nanocarrier encapsulating essential oils.

Silica nanocarriers encapsulating essential oils were synthesized by interfacial miniemulsion. In detail (Figure 1), the oil phase was prepared by mixing 2 g of TEOS precursor, 125 mg of MilliQ water and 1 g of EOs (LO, LO and CO, LO and TO). When used as mixtures, LO and CO, as well as LO and TO, were added in a 1:1 (w/w) ratio. Then 30 mL of 0.77% (w/w) aqueous solution of Triton X was added to the oil phase. The mixture was stirred with magnetic stirring for 10 min. Subsequently, the suspension was ultrasonicated (3 min, 70% amplitude in a pulsed regime with 30 s on and 10 s off, power output 90 W) in an ice bath with the consequent formation of miniemulsion. Finally, the miniemulsion was left to stir for 20 h at room temperature to complete the formation of SiO_2_@Essential Oil nanocarriers (Jobdeedamrong et al., 2018). The synthesis of the control sample (SiO_2_ ctrl) was performed in the same way but without the addition of essential oils.

### 2.3 Characterization techniques

#### 2.3.1 X-ray diffraction (XRD) analysis

The SiO_2_ nanocarrier, initially in suspension, was oven-dried at 100 °C for 3 h to yield a powder. The XRD pattern of SiO_2_ nanocarrier powder was obtained using a Bragg–Brentano diffractometer (Bruker D8 Advance, Karlsruhe, Germany) operating in a θ/2θ configuration, with an X’Celeretor detector LynkEye (5°–80°, 2θ range, 0.02 step size, 0.5 s per step).

#### 2.3.2 Colloidal characterization

Hydrodynamic diameter (d_DLS_) and Zeta Potential (ZP) were assessed for the synthesized nanocarriers by means of Dynamic Light Scattering (DLS) and Electrophoretic Light Scattering (ELS) techniques with a Zetasizer Nano instrument ZSP (model ZEN5600, Malvern Instruments, United Kingdom) (Bhattacharjee, 2016). The stock dispersions emerging from the encapsulation were diluted in deionized water to obtain a final concentration of 50 μg/mL. After a 1-min temperature equilibration step, the particle size distribution (nm) by intensity of 1 mL of sample was measured three times consecutively at 25 °C and was obtained by averaging the three measurements with relative standard deviations. After ascertaining the particle size, the samples underwent ZP measurement by electrophoretic light scattering (ELS). The ZP was measured on 700 µL of sample at 25 °C, with an automatic duration of the measurement, an attenuator position, and an applied voltage. After a 1-min temperature equilibration step, samples dispersed in deionized water were measured three times at 60 s intervals and ZP data were obtained by averaging the three measurements. The Smoluchowski equation was applied to convert the electrophoretic mobility to ZP. The ZP was also measured for the samples diluted (50 μg/mL) in the relevant media: 1% w/w Tween 80 solution in distilled water and Mueller–Hinton broth (MHB) for release and antibacterial tests, respectively.

#### 2.3.3 Fourier transform infrared spectroscopy

FTIR analysis was performed on silica nanocarrier encapsulating essential oils to ensure EOs loading. The measurements were obtained using a Nicolet iS5 spectrometer (Thermo Fisher Scientific Inc. - Waltham, MA, United States). Samples were analysed in ATR mode and in a wavenumber range between 400 and 4,000 cm^−1^. Spectra were collected with a resolution of 2 cm^−1^ and by means of 32 scans. The positions of the peaks were identified using the OMNIC software, and values were compared with the data in the literature (Boutagayout et al., 2025; Merve et al., 2023).

#### 2.3.4 Morphological characterization

Morphological characterization of the nanocarrier was performed by scanning transmission electron microscopy (STEM) analysis using a field emission-scanning electron microscopy (FE-SEM) instrument (Carl Zeiss Sigma NTS Gmbh, Oberkochen, DE). One drop of the suspension diluted to 100 mg/L in Milli-Q water was deposited on a film-coated copper grid and then dried in air.

#### 2.3.5 Specific surface area measurements and porosity characterization

The specific surface area, on SiO_2_ ctrl and SiO_2_@LO_CO samples, was measured by N_2_ physisorption apparatus (Surfer, Thermo Fisher Scientifc, Waltham, Massachusetts, United States) via Brunauer–Emmett–Teller (BET) analysis, in which samples were pre-treated under a vacuum (P < 4 mbar) for 2 h. Porosity and open pore size distribution, on SiO_2_@LO_CO sample, were analyzed by mercury intrusion porosimetry (MIP, Pascal 140 and Pascal 240 series, Thermo Finnigan, United States); the error is estimated to be ± 4%, according to the instrument specifications and calibration range.

#### 2.3.6 Thermogravimetric analysis

The thermogravimetric analysis (TGA), coupled with the differential scanning calorimetry analysis (DSC), of the nanocarrier was performed by the STA 449 F3 Jupiter instrument (NETZSCH Group - Selb, Germany). The analyses were conducted in air, using a mixture of N_2_ and O_2_ in the ratio 92:8 by volume and using a gas flow of 50 mL/min. All the samples were heated at 10 °C min^−1^ up to 800 °C (Berraaouan et al., 2023). The thermograms were acquired by measuring 10 points over °C and were analyzed using the *Proteus* software. The SiO_2_@EOs samples were obtained by centrifugation of nanocarrier suspension, gaining after 10 min at 15,300 rpm SiO_2_@EOs solid residues. They were collected and air-dried at 40 °C for 1 h. The solid residues were then analyzed by TGA analysis.

#### 2.3.7 High performance liquid chromatography (HPLC)

The loading capacity, encapsulation efficiency of essential oils in silica nanocarriers, and release kinetics were evaluated by HPLC (High Performances Liquid Chromatography, Agilent 1260 Infinity II, USA) (Garcia et al., 2026; Fan et al., 2015). Analyses were carried out using InfitityLab Poroshell 120 EC-C18 Column (4.6 × 100 mm, 2.7 µm, Agilent). The mobile phase consisted of a mixture of acetonitrile, water, and ethanol (suitable for GC analysis) with a gradient elution profile as detailed in Supplementary Table S1. The column temperature and the ultraviolet detector were set respectively at 5 °C and 210 and 220 nm. The quantitative composition of the EOs was experimentally determined using this HPLC method. Evaluation of the EOs content and release kinetics within silica nanocarriers was performed by analyzing key components. Linalool was specifically targeted for LO, cinnamaldehyde for CO, and thymol for TO. Each samples were made by three independent measurements with an injection volume of 20 µL.

##### 2.3.7.1 Loading capacity and encapsulation efficiency

The amount of encapsulated EOs was determined using HPLC (High Performance Liquid Chromatography, Agilent 1260 Infinity II, United States). The values were calculated following Equations 1 and 2, establishing the loading capacity (LC) and the encapsulation efficiency (EE), respectively (Giotopoulou et al., 2024).

$$LC=\frac{Weight\;of\;essential\;oil\;in\;nanocapsules}{Weight\;of\;nanocapsules\;}\times 100$$ (1)

$$EE=\frac{Weight\;of\;essential\;oil\;in\;nanocapsules}{Weight\;of\;essential\;oil\;added\;}\times 100$$ (2)

##### 2.3.7.2 EO release kinetics

The release of the EOs was investigated by analyzing their release kinetics, allowing us to characterize the rate by which the active compounds diffused from the nanocarriers over time (Berraaouan et al., 2023). Specifically, the SiO_2_@EOs (25 mg) were placed in a glass flask containing 3 mL of the release medium (1% w/w Tween 80 in distilled water). The suspensions were gently stirred at ambient temperature (20 °C). At predetermined time intervals (1, 2, 4, 6, 24, 48, 72, 96, and 168 h), samples were withdrawn. The procedure involved taking all aliquots from the release medium, which were then centrifuged at 15,300 rpm for 10 min. The supernatants were diluted in ethanol (dilution EtOH:SiO_2_@EOs, 1:4 for SiO_2_@LO and SiO_2_@LO_TO; 1:8 for SiO_2_@LO_CO) to ensure complete solubilization of the EOs and to keep the measurements within the linear range of the calibration curve. The supernatants were then analyzed by HPLC (Agilent 1260 Infinity II, United States). The release percentage is calculated relative to the effective loading of the specific batch used. Each time point was performed using independent samples, and all experiments were carried out in triplicate.

### 2.4 Evaluation of antibacterial activity

The antibacterial activity of free essential oils, their combinations, and silica-encapsulated formulations was evaluated by determining the minimum inhibitory concentration (MIC) against *Escherichia coli* ATCC 8739 and *Staphylococcus aureus* ATCC 6538P using a broth microdilution method.

For MIC determination of free essential oils, lavender oil (LO), cinnamon oil (CO) and thyme oil (TO) were first pre-treated following the procedure described by Van et al. (2022). Briefly, each essential oil was mixed with ultrapure water in a 1:1 (v/v) ratio and subjected to ultrasonication to promote dispersion. After sonication, the mixtures were allowed to stand, and the lower aqueous phase was carefully collected and used for subsequent MIC assays. Silica-encapsulated formulations (SiO_2_@LO, SiO_2_@LO_CO, and SiO_2_@LO_TO) and blank silica nanoparticles were directly dispersed in MHB containing 0.2% (v/v) Tween 80 to ensure homogeneous suspension.

Bacterial strains were grown overnight in Mueller–Hinton broth (MHB) at 37 °C under aerobic conditions. Each well was inoculated with the bacterial suspension to reach a final concentration of 1 × 10^6^ CFU/mL and incubated at 37 °C for 24 h. Serial two-fold dilutions of all samples were prepared in sterile 96-well microtiter plates to obtain final concentrations expressed as % (v/v). Growth controls (bacteria without treatment) were included in all experiments. Blank silica nanoparticles were tested under the same conditions. After incubation, bacterial growth was assessed by visual inspection and confirmed by serial dilutions and plating on solid media. The MIC was defined as the lowest concentration of the sample at which no bacterial growth was observed.

### 2.5 Statistical analysis

All experimental measurements, including colloidal characterization (DLS and ELS), HPLC, and TGA quantifications for loading capacity, encapsulation efficiency, and release kinetics, were performed in triplicate using independent samples ( n=3 ), and results are expressed as mean ± standard deviation (SD). Data processing and kinetic model fitting were performed using Microsoft Excel 365 (version 2606). The dimensional analysis, from FE-SEM images, was performed by means of ImageJ software on a total of 50 nanoparticles.

## 3 Results and discussion

### 3.1 Characterization of essential oils in silica nanocarriers

The encapsulation process of the EOs was carried out by an integrated aqueous sol–gel/interfacial miniemulsion strategy. In this configuration, the essential oil phase contributes to emulsion stabilization, allowing for nanocarrier formation without the need for organic co-solvents or cationic templating agents typically used in conventional protocols. The synthesis was conducted using water as the externally added solvent, TEOS as the silica precursor and the non-ionic surfactant Triton X-100 under controlled conditions (Figure 1), thereby reducing the use of additional organic co-solvents and simplifying the formulation chemistry compared with the alternative formulations investigated. This comparative design choice is consistent with an early-stage, SSbD-inspired hazard-reduction approach, although it does not constitute a complete quantitative SSbD assessment.

**FIGURE 2:**
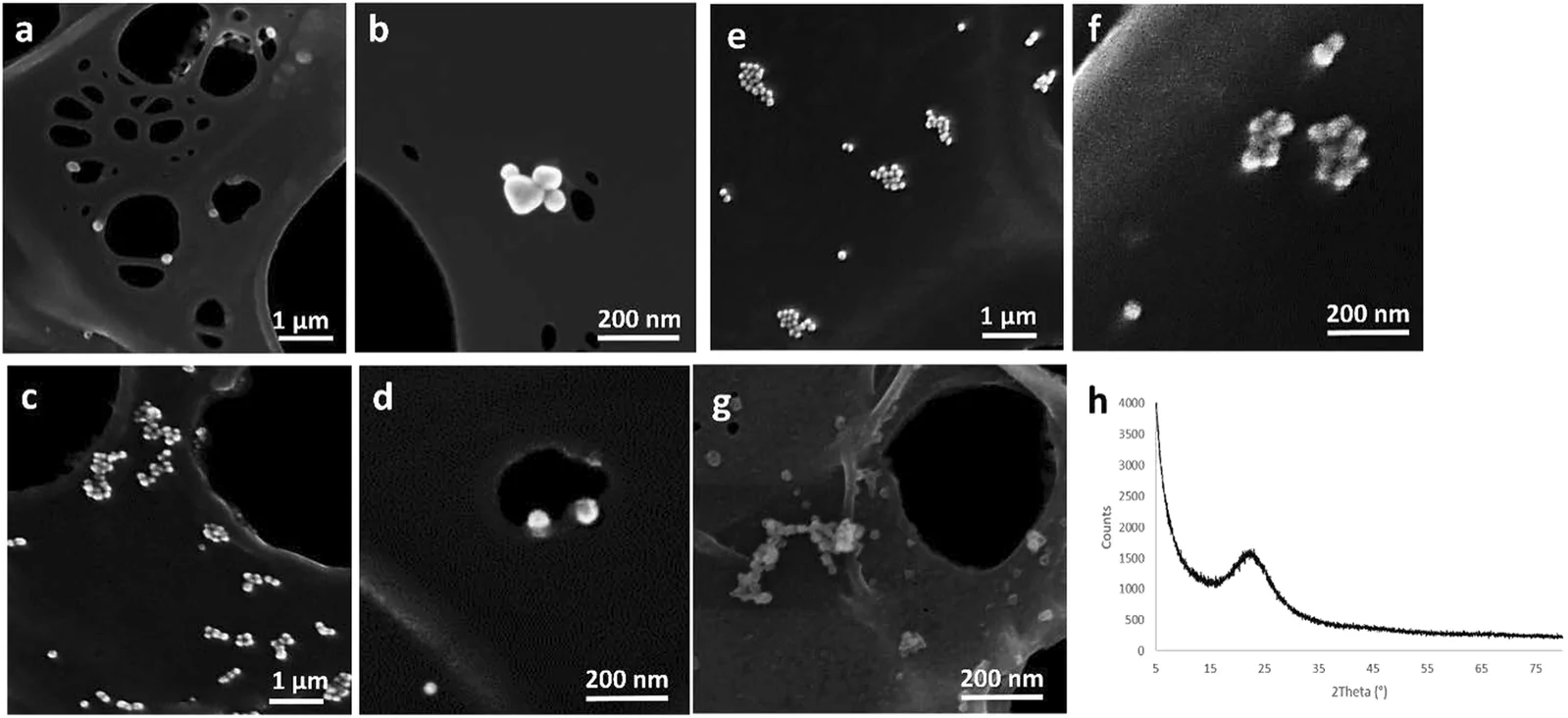
FESEM-STEM images of synthesized SiO_2_ nanocarriers: **(a,b)** SiO_2_@LO; **(c,d)** SiO_2_@LO_TO; **(e,f)** SiO_2_@LO_CO; **(g)** SiO_2_ ctrl and **(h)** X-ray diffractogram of SiO_2_ ctrl.

The successful formation of silica nanocarriers encapsulating LO and its blends with CO and TO was evaluated through complementary physicochemical techniques. These analyses allowed verification of the structural nature of the silica matrix, particle morphology, and size distribution, surface properties, and encapsulation efficiency. X-ray diffraction (XRD) analysis confirmed that the microemulsion-based synthesis yielded amorphous silica as the encapsulating phase. The pattern of SiO_2_ ctrl (Figure 2h) displays a broad diffraction band at 2θ = 22.0°, typical of amorphous SiO_2_. FESEM-STEM images (Figure 2) show well-dispersed SiO_2_ nanocarriers with a uniform spherical morphology across all formulations (SiO_2_@LO, SiO_2_@LO_TO, and SiO_2_@LO_CO), as well as SiO_2_ ctrl without the EOs. Particle size distribution evaluated by FESEM-STEM image analysis showed average diameters ranging from 55 to 80 nm, in good agreement with DLS measurements with average diameters ranging from 56 to 85 nm (Table 1), which confirms narrow size distributions and very low PdI values (<0.15), demonstrating high colloidal stability and monodispersity. Zeta potential analysis revealed negative surface charges for all formulations, consistent with the typical surface properties of SiO_2_ nanoparticles (isoelectric point < 1.5) (Faccani et al., 2021), and indicative of good colloidal stability. Although the absolute values of the zeta potential are not high enough to indicate excellent colloidal stability, in silica-based colloidal systems, high colloidal stability can be achieved even when the absolute zeta potential values are in the range of 10–15 mV (Bhattacharjee, 2016). Minor variations were observed depending on the incorporated essential oil; however, all systems maintained stable and well-defined nanostructures. Despite minor variations in hydrodynamic diameter and surface charge, all formulations maintained narrow size distributions and negative zeta potentials, confirming the successful formation of colloidally stable silica nanocarriers (Rodriguez-Loya et al., 2024). The SiO_2_ control sample exhibited a diameter of 146.1 ± 11.0 nm (d_DLS_), 139 ± 8 nm (d_SEM_) and a negative zeta potential of −10.3 ± 0.2 mV (pH = 6), indicating good colloidal stability, in agreement with the results obtained for the samples containing the EOs (Table 1).

**TABLE 1:** Colloidal characterization of encapsulated essential oils in SiO_2_ nanocarriers (lavender oil SiO_2_@LO, and the mix lavender + cinnamon SiO_2_@LO_CO and lavender + thyme SiO_2_@LO_TO). The values were obtained by averaging the three measurements with relative SD. The d_SEM_ was a mean of 50 nanoparticles.

Sample	d_DLS_ (nm)	d_SEM_ (nm)	PdI	ZP_ELS_ (mV)	pH
SiO_2_@LO	70.0 ± 0.3	73 ± 15	0.12	−18 ± 0.5	6
SiO_2_@LO_TO	85.3 ± 2.1	80 ± 10	0.15	−17 ± 0.7	6
SiO_2_@LO_CO	56.6 ± 2.0	55 ± 13	0.14	−15.5 ± 0.3	6
SiO_2_ ctrl	146.1 ± 11.0	139 ± 8	0.25	−10.3 ± 0.2	6

**FIGURE 3:**
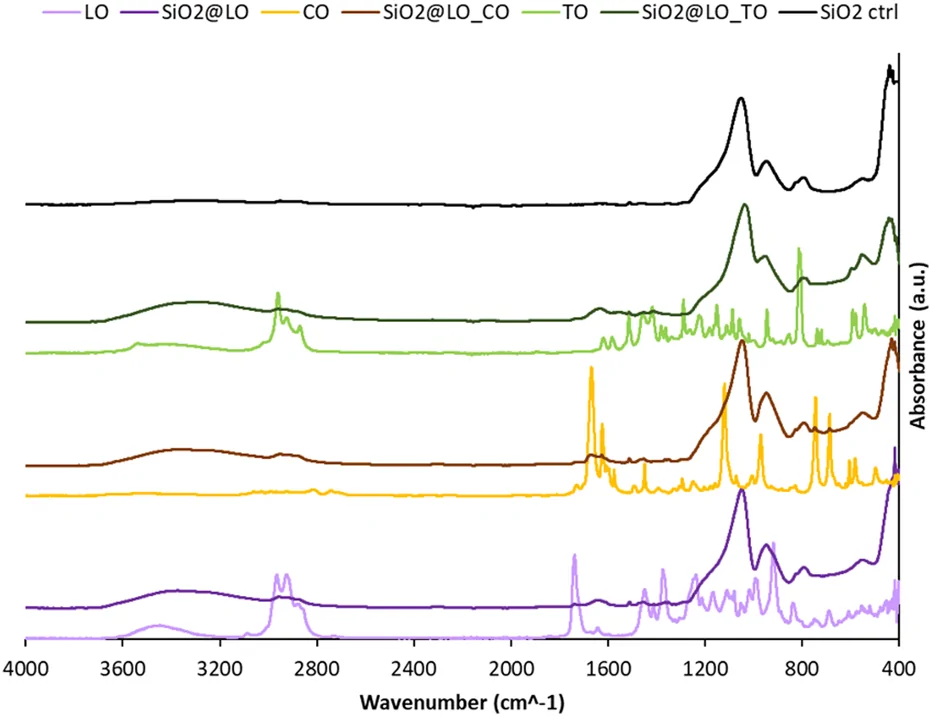
ATR-FTIR analysis of pure EOs and SiO_2_: LO, CO, TO, SiO_2_ ctrl and the nanocarriers: SiO_2_@LO, SiO_2_@LO_CO, SiO_2_@LO_TO.

Figure 3 reports the FTIR-ATR spectra used to verify the successful encapsulation of the essential oils within the silica nanocarriers. The spectrum of pure lavender oil (LO) shows characteristic absorption bands at 1740 cm^−1^ (ester C=O stretching of linalyl acetate), 1,644 cm^−1^ (C=C stretching), and intense aliphatic C–H stretching vibrations at 2962, 2923, and 2873 cm^−1^, consistent with the presence of linalool and linalyl acetate (Merve et al., 2023; Khatri et al., 2023). Cinnamon oil (CO) exhibits a characteristic aldehydic C=O stretching band at 1,667 cm^−1^ and aromatic ring vibrations around 1,623 cm^−1^, while thyme oil (TO) presents bands attributable to thymol, particularly in the region 806–942 cm^−1^ (aromatic C–H out-of-plane bending) and between 1,000 and 1,300 cm^−1^ (Giotopoulou et al., 2024). After encapsulation, the spectra of SiO_2_@EO nanocarriers retain the main characteristic bands of the respective essential oils, although with reduced intensity due to the dominant contribution of the silica matrix. In addition, a strong absorption band around 1,040–1,050 cm^−1^ corresponding to asymmetric Si–O–Si stretching confirms the formation of the silica network (Ishak et al., 2020), as shown by the SiO_2_ ctrl (black curve). The presence of–OH groups is evidenced by bands near 3,390 cm^−1^ and 936 cm^−1^, associated with residual silanol groups formed during TEOS hydrolysis. Overall, the coexistence of silica-related bands and characteristic EO peaks confirms the successful incorporation of the essential oils within the silica nanocarriers without significant alteration of their functional groups.

Thermogravimetric analysis (TGA) was performed to investigate the thermal behaviour of the pure essential oils and the corresponding SiO_2_@EO nanocarriers. The TGA profiles of LO, CO, and TO (Figure) showed distinct onset temperatures, reflecting differences in intrinsic volatility. CO exhibited the highest thermal stability (onset ≈ 140 °C), whereas LO and TO displayed lower onset temperatures (≈94 °C and 66 °C, respectively), in agreement with literature data (Hazra et al., 2002; Shlosman et al., 2024). Following encapsulation, the thermograms of the SiO_2_@EO samples (Supplementary Figure S1) revealed a characteristic mass-loss step associated with the volatilization of the incorporated oils superimposed on the thermally stable silica matrix. The residual mass at high temperature confirmed the presence of the inorganic SiO_2_ framework, while the relative weight loss allowed estimation of the encapsulated EO fraction, indicating efficient loading within the nanocarriers. HPLC analysis (Section 2.3.6) was used to determine the quantitative composition of the principal active constituents in the essential oils. The measured percentages were consistent with gas chromatography (GC) data reported in the literature (Table 2), confirming the expected composition of the oils before encapsulation. These results underscore that HPLC–UV, particularly when applied to the quantification of specific bioactive markers, represents a robust and highly competitive alternative to Gas chromatography (GC)-based methods, providing comparable quantitative accuracy while offering distinct advantages in terms of sample stability, selectivity, and compatibility with aqueous release studies (Garcia et al., 2026).

**TABLE 2:** Relative abundance (%) and retention times of major constituents in the essential oils determined by HPLC, compared with literature data.

Essential oils	Key constituent	Experimental content (%)	Literature content (%)	Retention time (min)**
Lavender	Linalool	38%	20%–45%*	9.9
Cinnamon	Cinnamaldehyde	75%	80%^^^	5.9
Thyme	Thymol	70%	77%^§^	11.7

*(Khatri et al., 2023), ^(Badr et al., 2022), ^§^(Ben Jabe et al., 2017), **Calibration curve and chromatogram reported in Supplementary Figures S2 and S3 of ESI.

### 3.2 Evaluation of loading capacity and encapsulation efficiency

**FIGURE 4:**
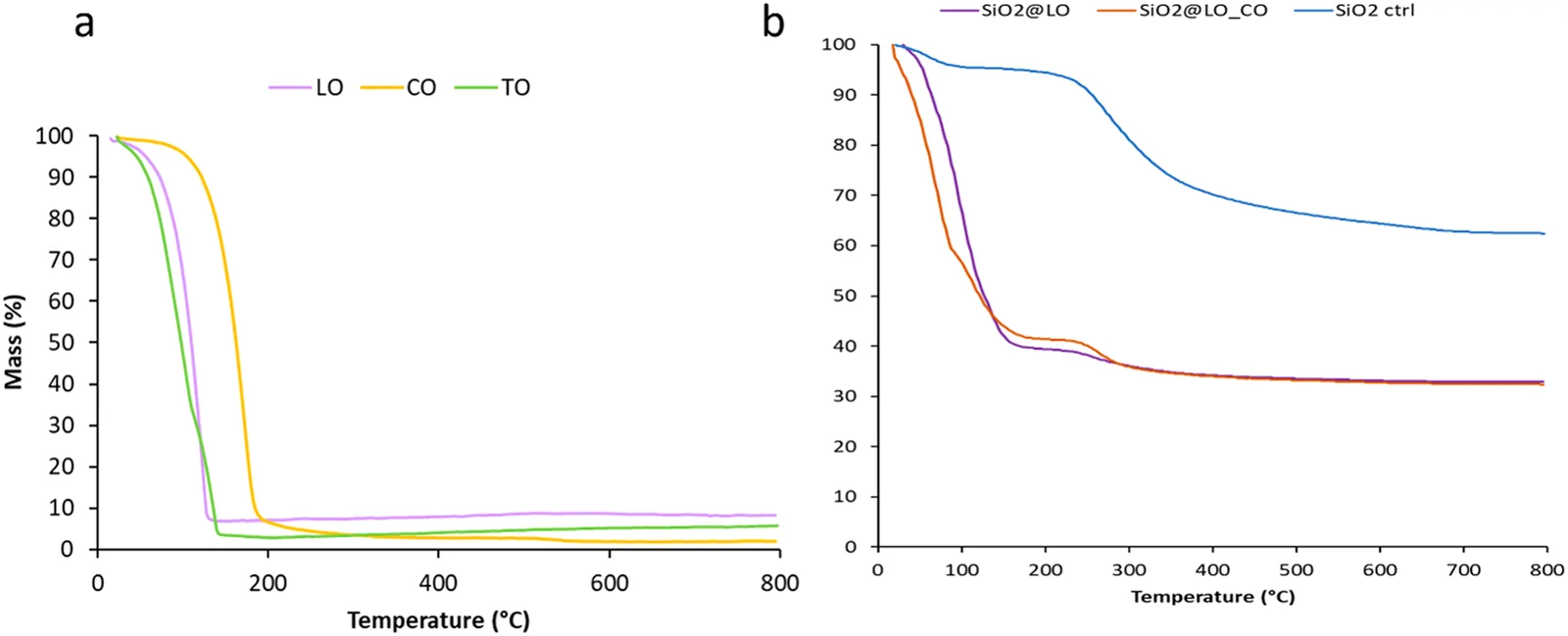
TG analysis of **(a)** EOs (LO: onset 94 °C, median 110 °C; CO: onset 140 °C, median 163 °C; TO: onset 66 °C, median 102 °C); **(b)** SiO_2_ ctrl, SiO_2_@LO, and SiO_2_@LO_CO. SiO_2_@LO_TO was unable to collect data.

Encapsulation performance was assessed by combining thermogravimetric analysis (TGA) and HPLC quantification. TGA was used to determine the EO content incorporated within the silica nanocarriers, whereas HPLC analysis quantified the residual non-encapsulated fraction in the supernatant (Table 3; Supplementary Tables S2–S4; Figure 4). The calibration curves and the chromatograms obtained by HPLC analysis are reported in Supplementary Figures S2 and S3. Encapsulation efficiency (EE%) was calculated by comparing the amount of EO encapsulated within the nanocarriers to the initial EO content used in the synthesis. HPLC analysis revealed minimal EO concentrations in the supernatant for all formulations (SiO_2_@LO, SiO_2_@LO_TO, and SiO_2_@LO_CO), corresponding to high EE% values and indicating efficient incorporation into the silica matrix. Loading capacity (LC%) was determined from the EO fraction present in the nanocarriers. For SiO_2_@LO, HPLC analysis yielded a loading of 54 wt%, in close agreement with the theoretical value (55.6 wt. %) and with the TGA-derived value (52 wt. %). For the mixed systems (SiO_2_@LO_TO and SiO_2_@LO_CO), the combined LC% values of the two essential oils reflected the nominal 1:1 composition introduced during synthesis, confirming controlled incorporation of both components. A comparison between TGA and HPLC data showed strong agreement, demonstrating the complementarity of the two techniques in evaluating encapsulation performance. While TGA provides a direct measure of total organic content, HPLC enables selective quantification of individual EO constituents, offering additional compositional insight. The consistency between the two methods supports the robustness and reliability of the encapsulation assessment. It should be noted that, for the SiO_2_@LO_TO sample, TGA quantification was not feasible due to post-centrifugation instability, a behaviour also observed during release kinetics studies.

**TABLE 3:** Encapsulation efficiency and loading capacity calculated by TGA and HPLC analysis on SiO_2_@EOs samples. The values were obtained by averaging the three measurements with relative SD.

Samples	TGA analysis		HPLC analysis					
	Encapsulation efficiency (EE%)	Loading capacity (LC%)	LO encapsulation efficiency (EE%)	LO loading capacity (LC%)	CO encapsulation efficiency (EE%)	CO loading capacity (LC%)	To encapsulation efficiency (EE%)	To loading capacity (LC%)
SiO_2_@LO	93 ± 1	52 ± 1	97 ± 1	54 ± 1	—	—	—	—
SiO_2_@LO_CO	94 ± 1	52 ± 1	>99	28 ± 1	93 ± 1	26 ± 1	—	—
SiO_2_@LO_TO	n.a*	n.a*	>99	28 ± 1	—	—	>99	28 ± 2

*Not applicable.

### 3.3 Release test results

**FIGURE 5:**
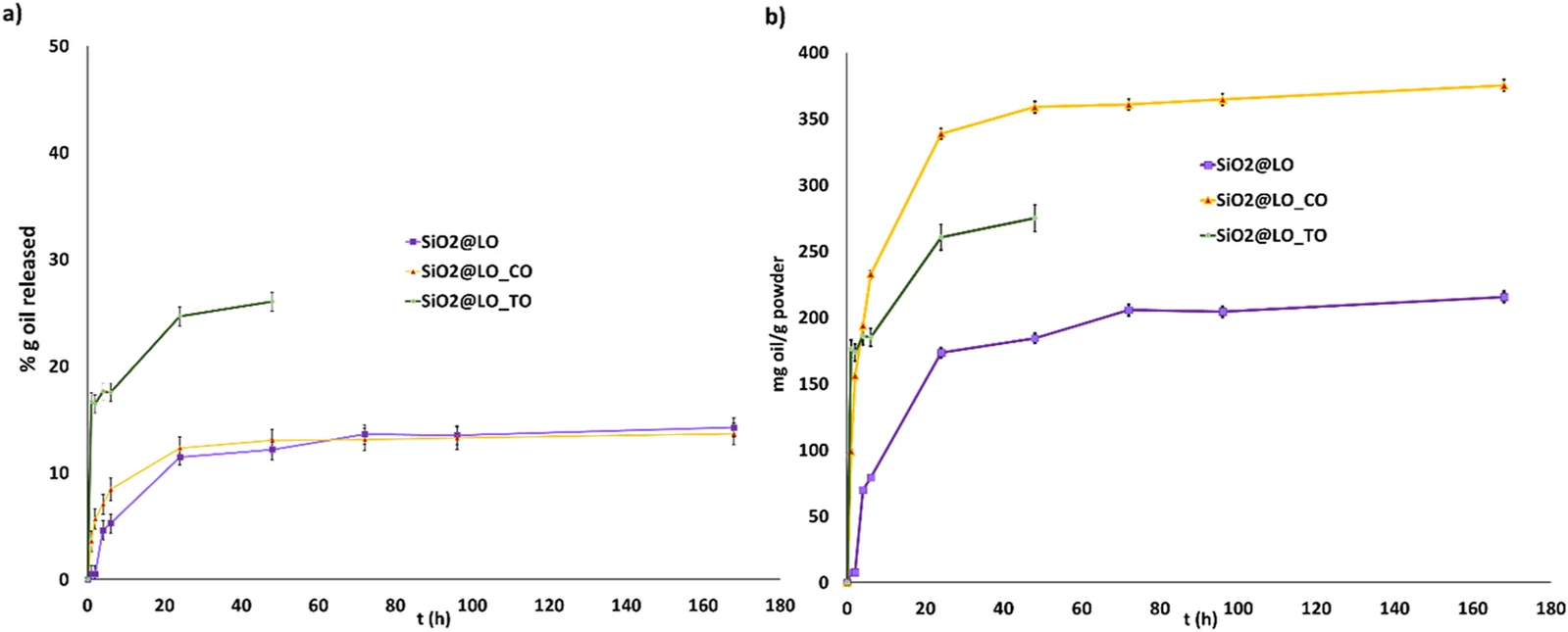
Release kinetic profile of SiO_2_@LO, SiO_2_@LO_TO, SiO_2_@LO_CO through HPLC analysis: **(a)** graph of percentage of grams of oil released; **(b)** graph of mg of oil released versus grams of initial powder. The values were obtained by averaging the three measurements with relative SD.

The release tests were carried out in the presence of 1% w/w Tween 80 solution in distilled water. Good colloidal stability of the SiO_2_ nanocarriers was also confirmed when dispersed in Tween 80 solution, as demonstrated by the zeta potential data reported in Supplementary Table S5. The release behaviour of the encapsulated essential oils was investigated by HPLC analysis (Figure 5). Distinct release profiles were observed depending on the oil composition and formulation. SiO_2_@LO and SiO_2_@LO_CO exhibited a gradual and limited release over time, reaching a maximum of approximately 15% of the encapsulated oil. In contrast, SiO_2_@LO_TO showed a faster and more pronounced release, with TO reaching 26% within 48 h. The accelerated release observed for TO suggests weaker interactions with the silica matrix compared to CO and LO, possibly due to differences in molecular structure and polarity. In mixed systems (SiO_2_@LO_CO and SiO_2_@LO_TO), only CO and TO were quantitatively detected in the release medium, as LO concentrations remained below the instrumental detection limit. This behaviour may indicate stronger retention of LO within the silica network, potentially related to specific interactions with silanol groups; however, further mechanistic investigation would be required to confirm this hypothesis. When expressed as mass of oil released per gram of initial sample (Figure 5b), the formulations displayed consistent trends. SiO_2_@LO showed the lowest cumulative release (≈215 mg g^−1^), whereas SiO_2_@LO_CO reached approximately 375 mg g^−1^ (see Supplementary Table S6). SiO_2_@LO_TO exhibited a rapid initial release within the first hour, followed by a plateau and a subsequent increase up to ≈ 275 mg g^−1^ at 48 h. It should be noted that release experiments for SiO_2_@LO_TO were limited to 48 h due to post-centrifugation instability of the nanocarriers, consistent with the lower structural stability observed for this formulation. Based on the release behavior, the most promising sample, SiO_2_@LO_CO, was selected for porosity and surface area analysis. The data revealed a specific surface area of 89.7 m^2^/g, and a porosity of 29%, with an average pore size of 0.050 µm (50 nm) and a modal pore size of 0.014 µm (14 nm). Although lower than that of highly open mesoporous frameworks, the specific surface area is consistent with the material’s dense structure, providing sufficient surface for essential oil interaction without compromising matrix integrity (Veith et al., 2005), and is comparable to that of the SiO_2_ ctrl (119 m^2^/g). Concurrently, the porosity percentage (<30%) indicates the formation of a relatively dense and robust silica framework. Such a compact matrix is highly desirable for the encapsulation of volatile compounds, as it provides an effective physical barrier that enhances the thermal and oxidative stability of the entrapped essential oils (Ashraf et al., 2015). Furthermore, the pore size distribution falls predominantly within the mesoporous range (2–50 nm). From a controlled-release perspective, this unique mesoporous architecture is highly advantageous. As demonstrated by the HPLC analysis (Figure 5), it successfully promotes a sustained, diffusion-controlled release profile over time, a behavior consistent with typical behavior of mesoporous silica systems (Zhong et al., 2021; Niroumand et al., 2023).

In addition to the experimental release profiles, the data were fitted to four kinetic models (Zero-order, First-order, Higuchi, and Korsmeyer–Peppas) to elucidate the predominant transport mechanism (Table 4). The Zero-order model showed poor correlation for all systems, indicating that release does not proceed at a constant rate and is not governed by surface erosion. The First-order model provided the highest correlation coefficients for SiO_2_@LO and SiO_2_@LO_CO (*R* ^2^ = 0.9926 and 0.9915, respectively), demonstrating that the release rate strongly depends on the residual oil concentration within the silica matrix. This behavior suggests that concentration-driven desorption plays a dominant role in these two systems (Zhang et al., 2024). The Higuchi model yielded moderate correlation coefficients (*R* ^2^ = 0.82–0.92), confirming that diffusion through the relatively porous silica structure contributes significantly to the release process, although it does not fully capture the overall kinetics. However, since it is not the best-fitting model, diffusion cannot be considered purely Higuchian (Xu et al., 2023). The Korsmeyer–Peppas model did not outperform the First-order model for SiO_2_@LO and SiO_2_@LO_CO; however, it provided good fits across all systems (*R* ^2^ = 0.95–0.99) and enabled evaluation of the diffusion exponent n. For all formulations, n was below 0.45 (SiO_2_@LO: 0.34; SiO_2_@LO_CO: 0.19; SiO_2_@LO_TO: 0.15), which is indicative of Fickian diffusion in rigid, non-swelling matrices. Therefore, although the First-order model best describes the release kinetics of SiO_2_@LO and SiO_2_@LO_CO, the n values obtained from the Korsmeyer–Peppas equation confirm that diffusion through the silica network represents the underlying transport mechanism (Wang et al., 2024).

**TABLE 4:** Fitting results of the release kinetic profile of SiO_2_@LO, SiO_2_@LO_TO, SiO_2_@LO_CO.

Model release equation	SiO_2_@LO	SiO_2_@LO_CO	SiO_2_@LO_TO
Zero-order kinetic	y = 0.085 x *t* *R* ^2^ = 0.7946	y = 0.062 x *t* *R* ^2^ = 0.7162	y = 0.32 x *t* *R* ^2^ = 0.6864
First-order kinetic	y = 13.53 x (1-e^−0.077t^) *R* ^2^ = 0.9926	y = 12.57 x (1-e^−0.172t^) *R* ^2^ = 0.9915	y = 13.53 x (1-e^−15.20t^) *R* ^2^ = 0.8852
Higuchi kinetic	y = 1.41 x t^1/2^ *R* ^2^ = 0.9219	y = 1.46 x t^1/2^ *R* ^2^ = 0.8703	y = 0.99 x t^1/2^ *R* ^2^ = 0.8264
Korsmeyer-Peppas kinetic	y = 2.83 x t^0.34^ *R* ^2^ = 0.9501	y = 5.49 x t^0.19^ *R* ^2^ = 0.9701	y = 14.47 x t^0.15^ *R* ^2^ = 0.9905

Overall, the release profiles exhibited an initial faster phase followed by stabilization after 24 h, with a maximum release of 26% observed for SiO_2_@LO_TO at 48 h SiO_2_@LO showed the slowest and most limited release, suggesting stronger retention of LO within the silica matrix. In mixed systems, LO concentrations in the release medium remained below the detection limit, which may indicate preferential retention within the nanocarriers, although further investigation would be required to clarify the interaction mechanisms involved. The SiO_2_@LO_TO formulation displayed reduced structural stability beyond 48 h, preventing extended release analysis and suggesting weaker matrix integrity in this system. Taken together, the kinetic modelling results indicate that essential-oil release is primarily governed by diffusion through the silica shell, while the best-fitting kinetic model depends on the specific formulation. The First-order model is dominant for SiO_2_@LO and SiO_2_@LO_CO, whereas SiO_2_@LO_TO follows the Korsmeyer–Peppas model more closely. The diffusion exponent obtained from the Korsmeyer–Peppas equation consistently supports a Fickian diffusion mechanism across all systems.

### 3.4 Antibacterial activity

The antibacterial activity of free essential oils, their binary mixtures, and the corresponding silica-encapsulated formulations was evaluated against *Escherichia coli* ATCC 8739 and *S. aureus* ATCC 6538P. These two strains were selected as representative Gram-negative and Gram-positive bacterial strains, respectively, as they are widely used as reference bacteria for evaluating the antibacterial activity of new bioactive materials and formulations. Minimum inhibitory concentration (MIC) values are summarized in Table 5. Among the individual oils, lavender oil (LO) exhibited negligible antibacterial activity against both strains (MIC > 50% v/v), indicating its limited efficacy when used alone. Cinnamon oil (CO) demonstrated the strongest activity, particularly against *E. coli*, with MIC values of 1.56% and 3.12% against *Staphylococcus aureus*, consistent with the well-documented antimicrobial role of cinnamaldehyde (Jaramillo Jimenez et al., 2024). Thyme oil (TO) showed moderate antibacterial effects, with MIC values of 12.5% for *E. coli* and 6.25% for *S. aureus*, in agreement with the activity typically associated with thymol-rich oils (Fathy et al., 2025). The use of essential oil blends was explored to evaluate whether the combination of LO with other essential oils (TO and CO) could provide complementary and synergistic effects. Binary combinations exhibited different antibacterial behaviors. The LO_CO mixture showed antibacterial activity comparable to cinnamon oil against *S. aureus*, but no improvement over cinnamon oil against *E. coli*. In contrast, the LO_TO mixture displayed lower MIC values than thyme oil alone against both bacterial strains. This latter result also suggests that lavender oil can provide antibacterial performance when combined with thyme oil. This observation is consistent with previous studies reporting complementary antibacterial mechanisms, including membrane disruption and increased permeability (Spada et al., 2021).

**TABLE 5:** Minimum inhibitory concentration (MIC) values, expressed as % (v/v), of free and silica-encapsulated essential oils and their combinations against *Escherichia coli* ATCC 8739 and *Staphylococcus aureus* ATCC 6538P.

Samples	*E. coli* ATCC 8739	*S. aureus* ATCC 6538P
LO	>50	>50
CO	1.56	3.12
TO	12.5	6.25
LO_CO	3.12	3.12
LO_TO	3.12	3.12
SiO_2_ ctrl	>50	50.00
SiO_2_@LO	25.00	3.12
SiO_2_@LO_CO	6.25	0.78
SiO_2_@LO_TO	6.25	1.56

The colloidal stability of the SiO_2_ nanocarriers was studied in MHB. The data indicated that the samples retained their colloidal stability (Supplementary Table S5). The MIC values evaluating the antibacterial activity of the SiO_2_ nanocarriers are summarized in Table 5. Blank silica nanoparticles showed no detectable antibacterial effect. SiO_2_@LO retained antibacterial activity, with MIC values reduced to 25% for *E. coli* and 3.125% for *S. aureus*, indicating that encapsulation effectively preserved the antibacterial functionality. For mixed systems, SiO_2_@LO_CO showed MIC values of 6.25% against *E. coli* and 0.78% against *S. aureus*, while SiO_2_@LO_TO exhibited MIC values of 6.25% and 1.56%, respectively. Notably, the oil blends improved LO performance also within the encapsulated system. The different antibacterial responses observed for *E. coli* and *S. aureus* can be attributed, at least in part, to their different cell envelope composition and structure. As a gram-negative bacterium, *E. coli* possesses an outer membrane rich in lipopolysaccharides that acts as an additional permeability barrier, reducing the penetration of hydrophobic compounds such as essential oil constituents. In contrast, gram-positive *S. aureus* cells are composed of a thick peptidoglycan layer but lack an outer membrane, which may facilitate the interaction of encapsulated essential oil formulations with the cell membrane, leading to disruption of membrane integrity and cellular functions (Nazzaro et al., 2013; Ruiz-Rico et al., 2017).

In summary, the comprehensive physicochemical, release, and biological evaluations confirmed the successful development of silica nanocarriers through an SSbD-inspired, fully aqueous sol–gel/interfacial miniemulsion approach. The mesoporous SiO_2_ matrix effectively protected the essential oils, overcoming their volatility while enabling sustained Fickian release and preserving the integrity of the encapsulated compounds. Furthermore, co-encapsulation maintained the synergistic interactions of the essential oil blends, resulting in enhanced antibacterial activity against both *E. coli* and *S. aureus*.

## 4 Conclusion

Silica nanocarriers encapsulating individual lavender oil (LO) and binary synergistic blends of LO with cinnamon oil (CO) or thyme oil (TO) were successfully synthesized through a water-based sol–gel process combined with interfacial miniemulsion. The formulation was selected according to an early-stage, SSbD-inspired comparative screening of the investigated alternatives. Comprehensive characterization confirmed the formation of well-dispersed, spherical amorphous silica nanocarriers with uniform hydrodynamic diameters ranging from 55 to 85 nm and narrow size distributions (PdI < 0.15). Combined TGA and HPLC analyses validated the robustness of the encapsulation framework, demonstrating high encapsulation efficiencies of 93%–94% and a remarkable loading capacity of 52 wt. %.

Release kinetics studies quantified a sustained, formulation-dependent behavior driven by Fickian diffusion through the rigid silica shell. Cumulative release was limited to 15% over 168 h for SiO_2_@LO and SiO_2_@LO_CO, whereas SiO_2_@LO_TO exhibited a faster release, reaching 26% within 48 h due to weaker matrix interactions and post-centrifugation instability.

Antibacterial assays against *Escherichia coli* and *Staphylococcus aureus* showed that encapsulated EO blends preserved their core antibacterial activity. SiO_2_ nanocarriers loaded with LO showed MIC values to 25% for *E. coli* and 3.125% for *S. aureus*. Encapsulated mixed showed MIC values of 6.25% and 0.78% (for LO and CO), and 6.25% and 1.56% (for LO and TO) against *E. coli* and *S. aureus*, respectively, demonstrating that the oil blends improved LO performance also within the encapsulated system.

The present results demonstrate that this eco-friendly nanocarrier platform successfully tackles the volatility and stability limitations of essential oils, opening up promising and concrete applications for these sustainable formulations in biomedicine and the construction sector. Most notably, this system holds significant potential in the field of cultural heritage conservation, where these encapsulated formulations can be directly applied as eco-friendly biocides to counteract the biodeterioration of historical materials and stone surfaces, ensuring long-term protection without environmental hazards.

## Data Availability

The original contributions presented in the study are included in the article/Supplementary Material, further inquiries can be directed to the corresponding author.
