# Supplementary material for: Eco-friendly formulations of essential oil-based silica nanocarriers as antibacterial agents
Source: Front Bioeng Biotechnol. 2026 Aug 28;14:1903269. doi: 10.3389/fbioe.2026.1903269 (PMC13561984; doi:10.3389/fbioe.2026.1903269)
Supplement: Supplementary file 1 [file Supplementaryfile1.docx]

Eco-Friendly Formulations of Essential Oil-Based Silica Nanocarriers as Antibacterial Agents

Chiara Artusi ‡^1^, Ilaria Zanoni ‡^1^, Daniele Ghezzi^2^, Gabriela Graziani^3^, Marina Serantoni^1^, Magda Blosi^1^, Stefano Fedi^2^, Martina Cappelletti^2^, Anna Luisa Costa^1^, Enrico Sassoni^4^, Simona Ortelli^1*^

^1^CNR-ISSMC, National Research Council - Institute of Science, Technology and Sustainability for Ceramics, Via Granarolo 64, 48018 Faenza (RA), Italy

^2^ Department of Pharmacy and Biotechnology, University of Bologna, Via Irnerio 42, 40126 Bologna (BO), Italy

^3^Department of Human Sciences and Quality of life, San Raffaele Rome University, via di Val Cannuta 247, 00166 Rome, Italy

^4^DICAM, Department of Civil, Chemical, Environmental and Materials Engineering, University of Bologna, Via Terracini 28, 40131 Bologna (BO), Italy

‡These authors share first authorship

^*^ correspoding author: simoma.ortelli@issmc.cnr.it

Table S1: Mobile phase gradient conditions. A = H_2_O, B = ACN, C = EtOH.

| Time (min) | A (%) | B (%) | C (%) | Flow (mL/min) |
| --- | --- | --- | --- | --- |
| 0 | 70 | 30 | 0 | 1.0 |
| 12 | 45 | 55 | 0 | 1.0 |
| 13 | 45 | 55 | 0 | 1.0 |
| 14 | 0 | 0 | 100 | 0.8 |
| 26 | 0 | 0 | 100 | 0.8 |
| 28 | 70 | 30 | 0 | 1.0 |

Table S2: HPLC analysis on nanocarriers referred to LO. The values were obtained by averaging the three measurements with relative standard deviations.

| Samples | LO in the supernatant (mg/mL) | LO in nanocapsule (mg/mL) |
| --- | --- | --- |
| SiO_2_@LO | 0.42 ± 0.04 | 12.17 ± 0.12 |
| SiO_2_@LO_TO | * | 6.30 ± 0.63 |
| SiO_2_@LO_CO | * | 6.30 ± 0.63 |

* under limit of quantification (LOQ < 0.015 mg/mL) and detection (LOD < 0.05 mg/mL) of HPLC.

Table S3: HPLC analysis on nanocarriers referred to CO. The values were obtained by averaging the three measurements with relative standard deviations.

| Sample | CO in the supernatant (mg/mL) | CO in nanocapsule (mg/mL) |
| --- | --- | --- |
| SiO_2_@LO_CO | 1.60 ± 0.32 | 21.30 ± 0.42 |

Table S4: HPLC analysis on nanocarriers referred to TO. The values were obtained by averaging the three measurements with relative standard deviations.

| Sample | TO in the supernatant (mg/mL) | TO in nanocapsule (mg/mL) |
| --- | --- | --- |
| SiO_2_@LO_TO | * | 8.80 ± 0.35 |

* under limit of quantification (LOQ < 0.015 mg/mL) and detection (LOD < 0.05 mg/mL) of HPLC.

*Table S5: Zeta potential (ZP_ELS_) of SiO_2_ nanocarriers in the relevant media: 1% w/w Tween 80 solution in distilled water and Mueller–Hinton broth (MHB).*

| **Sample** | **ZP_ELS_ (mV)** | **pH** |
| --- | --- | --- |
| **Tween 80** | +10.6 ± 2.6 | 5 |
| **SiO_2_@LO in Tween 80** | +11.6 ± 0.3 | 5 |
| **SiO_2_@LO_CO in Tween 80** | +11.7 ± 0.9 | 5 |
| **SiO_2_@LO_TO in Tween 80** | +10.4 ± 0.6 | 5 |
| **MHB** | -4.3 ± 0.5 | 7 |
| **SiO_2_@LO in MHB** | -12.2 ± 0.8 | 7 |
| **SiO_2_@LO_CO in MHB** | -16.8 ± 1.9 | 7 |
| **SiO_2_@LO_TO in MHB** | -17.9 ± 1.3 | 7 |

The results indicated that the samples retained their colloidal stability in both media. In particular, the zeta potential values of the samples were comparable to those of the corresponding media alone, indicating that the media did not significantly affect their colloidal behavior.

*Table S6: Total encapsulation concentrations of the individual active compounds for each synthesis.*

| **Sample** | **Total Linalool encapsulation concentration (g/L)** | **Total Cinnamaldehyde encapsulation concentration (g/L)** | **Total Thymol**  **encapsulation concentration**  **(g/L)** |
| --- | --- | --- | --- |
| **SiO_2_@LO** | 12.6 | - | - |
| **SiO_2_@LO_TO** | 6.3* | - | 8.8 |
| **SiO_2_@LO_CO** | 6.3* | 22.9 | - |

* in SiO_2_@LO_CO and SiO_2_@LO_TO syntheses, linalool amount for half of the total quantity (6.3 g/L).

Figure S1: TGA analysis of Triton X-100 alone and SiO_2_ control.

*Figure S2: Calibration curves for each individual marker component of the essential oils quantified by HPLC analysis: a) Linalool b) Cinnamaldehyde c) Thymol.*

*Figure S3: Chromatograms of essential oils and the SiO_2_ control dispersed in Tween 80 solution obtained by HPLC analysis: a) Lavender oil b) Cinnamon oil c) Thyme oil d) Blank control SiO_2_ without essential oil*
